# Reviewing the Computational Landscape of Drug Repurposing: Evolution from Structure-Based Methods to LLM-Based Methods

**DOI:** 10.3390/biom16060830

**Published:** 2026-06-03

**Authors:** Zengyun Mou, Zhiqing Tian, Jiaqi Jin, Heng Yu, Yongzhen Huang

**Affiliations:** School of Artificial Intelligence, Beijing Normal University, Beijing 100088, China; mouzengyun@mail.bnu.edu.cn (Z.M.); 202521081036@mail.bnu.edu.cn (Z.T.); jinjiaqi@mail.bnu.edu.cn (J.J.); hengyu@bnu.edu.cn (H.Y.)

**Keywords:** drug repurposing, computational methods, Large Language Models (LLMs), review

## Abstract

Traditional drug discovery is a high-risk, time-consuming, and costly endeavor. Drug repurposing has emerged as a pivotal strategy to overcome these challenges by identifying new therapeutic indications for approved drugs, thereby significantly reducing development timelines, costs, and safety risks. This review aims to provide a comprehensive methodological survey of computational strategies for drug repurposing. It seeks to clarify the core principles, applicability, and limitations of various approaches, offering a clear technological landscape and valuable insights for future research directions. We categorize and elaborate on the prevailing methodologies, following a logical progression. The review begins with biological mechanism-driven methods, including structure-based, omics-based, fuzzy logic-based, and adverse event-based methods. It then details network-based methods that integrate multi-source data, encompassing graph mining and matrix factorization/completion techniques. Finally, we explore data-driven paradigms, tracing the evolution from traditional text mining-based methods to cutting-edge large language model (LLM)-based methods. Each methodological category presents unique advantages and challenges. While structure-based, omics-based, fuzzy logic-based, and adverse event-based methods provide deep mechanistic insights, network-based methods enable systematic prediction. Text mining unlocks information from vast literature, a potential greatly amplified by LLMs. This review highlights that the future of drug repurposing lies in the intelligent integration of diverse methodologies. In the future, we believe that network-based methods and data-driven methods will mark the beginning of large-scale drug repurposing, but ultimately, biological mechanism-driven methods will still be necessary for rigorous validation and explanation.

## 1. Introduction

Traditional drug discovery is a high-risk, high-cost endeavor [1] involving multiple stages: discovery and preclinical, safety review, clinical research, FDA review, and FDA post-market safety monitoring. This process is notoriously time-consuming, often exceeding a decade, and prohibitively expensive, with costs frequently surpassing billions of dollars, while facing a low probability of success [2]. To address these challenges, drug repurposing (also known as drug repositioning) has emerged as a promising strategy. It aims to identify new therapeutic indications for already-approved drugs [3]. Since these approved drugs have passed safety tests from prior use, drug repurposing can significantly bypass early-stage development hurdles, thereby drastically reducing the time, cost, and risk associated with bringing a new therapy to market, and accelerating the response to unmet medical needs [4].

A systematic review and summary of the methodologies employed in drug repurposing research are crucial for advancing the field. Therefore, this review, from a methodological perspective, aims to comprehensively survey existing computational strategies for drug repurposing, clarify the core principles, applicability, and limitations of different methods, and provide researchers with a clear technological landscape and valuable insights for future directions.

To ensure a coherent structure, this review is organized according to the intrinsic logic and evolution of the methodologies. It begins with an introduction to traditional methods focused on intrinsic biological mechanisms, primarily covering structure-based, omics-based, fuzzy logic-based and adverse event-based methods. It then elaborates on network-based methods that integrate multi-source heterogeneous data. Finally, it explores the progression from traditional text mining-based methods to large language model (LLM)-based methods, reflecting the latest data-driven research paradigms. The LLM-based methods excel at integrating multi-source data and capturing deep semantic and contextual information, but researchers should use them with caution.

**Figure 1:**
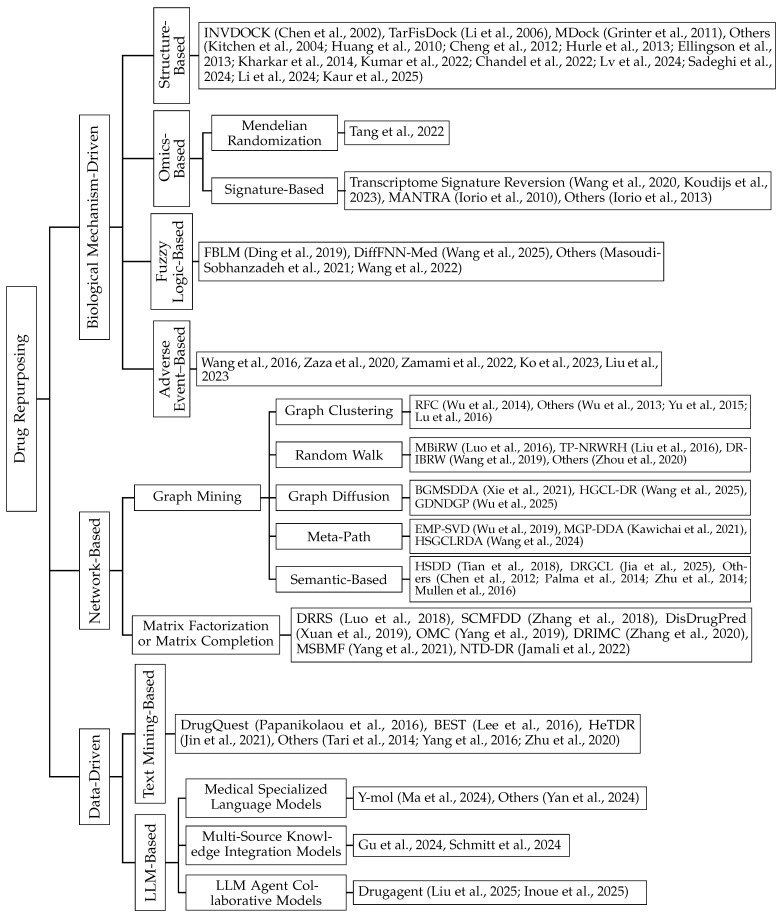
Taxonomy of drug repurposing. Biological mechanism-driven paradigms include structure-based, omics-based, fuzzy logic-based and adverse event-based methods; data-driven paradigms include text mining-based and LLM-based methods. The categories are partially overlapping and reflect the primary methodological orientation of each method. These studies are treated as preliminary evidence and are distinguished from peer-reviewed publications. References used in the taxonomy include: [5,6,7,8,9,10,11,12,13,14,15,16,17,18,19] (Structure-Based), [20,21,22,23,24] (Omics-Based), [25,26,27,28] (Fuzzy Logic-Based), [29,30,31,32,33] (Adverse Event-Based), [34,35,36,37,38,39,40,41,42,43,44,45,46,47,48,49,50,51,52,53] (Graph Mining), [54,55,56,57,58,59,60] (Matrix Factorization/Completion), [61,62,63,64,65,66] (Text-Mining-Based), [67,68,69,70,71,72] (LLM-Based).

The taxonomy of drug repurposing in our review is illustrated in Figure 1. The comparison of different computational methods is shown in Table 1. Our taxonomy is organized according to the core methodological principles underlying different drug repurposing methods, rather than being strictly defined by input data modalities or specific algorithmic families. This is because different methods may share overlapping data sources or algorithmic components. Accordingly, the categories are partially overlapping and are intended to provide a methodological orientation rather than strict mutual exclusivity. This is a methodological taxonomy rather than a strict chronological evolution.

This review proceeds by introducing the key methodological paradigms in drug repurposing. We begin with a brief overview of structure-based (Section 2.1.1) omics-based (Section 2.1.2), fuzzy logic-based (Section 2.1.3) and adverse event-based (Section 2.1.4) methods. Then, we provide a detailed discussion of network-based methods (Section 2.2), dividing the discussion into graph mining and matrix factorization/completion. Next, we introduce text mining-based methods (Section 2.3.1), which serve as a foundation for understanding the cutting-edge LLM-based methods detailed in Section 2.3.2 and Section 2.3.3. Then, we compare the data sources, evaluation metrics and validation strategies for various methods in Section 3. Finally, we discuss the future improvement directions for LLM-based methods and the future paradigm for drug repurposing in Section 4.1 and Section 4.2.

### Search Strategy and Selection Criteria

This review is developed through a staged literature search conducted up to January 2026. We first survey recent drug repurposing reviews [3,4] and examine the primary studies cited therein to establish an initial methodological framework [74,76,77,78,79,80,81,82]. Based on this framework, we then search PubMed and other biomedical literature resources, including publisher platforms such as MDPI and other publisher platforms as well as, where applicable, broader scholarly search engines and preprint servers, using combinations of “drug repurposing” or “drug repositioning” with method-specific terms such as “network-based”, “graph mining”, “matrix factorization”, “text mining”, “semantic”, “structure-based”, “omics-based”, and “fuzzy logic”. We additionally screen reference lists of relevant reviews and retrieve papers to identify representative studies that might otherwise have been missed. Non-English articles are excluded, and preprints are considered only when they report clearly original and methodologically relevant work and have not yet been superseded by a peer-reviewed publication. Study inclusion is guided primarily by methodological relevance, novelty, and representativeness within each method family. Notably, preprints are included only when no peer-reviewed alternative is available and are discussed with appropriate caution.

## 2. Methods

### 2.1. Biological Mechanism-Driven

#### 2.1.1. Structure-Based

Structure-based methods employ computational techniques to predict drug–target interactions, with molecular docking and molecular dynamics (MD) simulations as complementary approaches [15,18,19]. Molecular docking predicts ligand binding orientation and affinity within protein active sites using scoring functions that quantify non-covalent interactions [8,10]. Reverse docking screens a single compound against target libraries to identify novel indications or off-target effects [6,83]. MD simulations provide dynamic validation by assessing complex stability, capturing protein conformational changes, and revealing transient interactions in near-physiological conditions.

**Figure 2:**
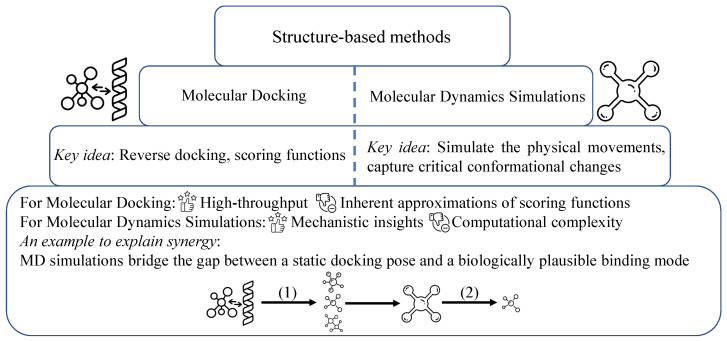
The synergy between molecular docking and MD simulations can be divided into: (1) Molecular Docking ranks potential drug candidates; (2) Molecular Dynamics Simulations verify the stability of the top-ranked complexes and uncover critical interactions, like hydrogen bonds.

The synergy between these methods is demonstrated through integrated pipelines, as illustrated in Figure 2. A study identifying novel MAO-B inhibitors combined pharmacophore screening with molecular docking, followed by MD simulations that confirmed dynamic stability and revealed additional hydrogen bonds with catalytic residues [14]. Similarly, a drug repurposing study for α-glucosidase inhibitors used molecular docking to prioritize DrugBank candidates, and then validated complex stability through 100-ns MD simulations and secondary structure analysis to explain inhibitory activity [17]. For broader applications, reverse docking platforms like TarFisDock and MDock screen compounds against extensive target libraries, successfully linking drugs like PRIMA-1 and torcetrapib to new targets and toxicity pathways [83,84].

The principal advantages include atomic-level mechanistic insights and cost-effective candidate prioritization. However, challenges remain: dependence on high-quality 3D target structures, substantial computational demands, and scoring function approximations [16]. Current solutions involve consensus scoring methods [9], hybrid approaches integrating structural and omics data [11,13], and enhanced computational capabilities. Despite these challenges, the integrated use of molecular docking and MD simulations has established itself as a powerful, rational strategy in modern drug repurposing.

#### 2.1.2. Omics-Based

**Figure 3:**
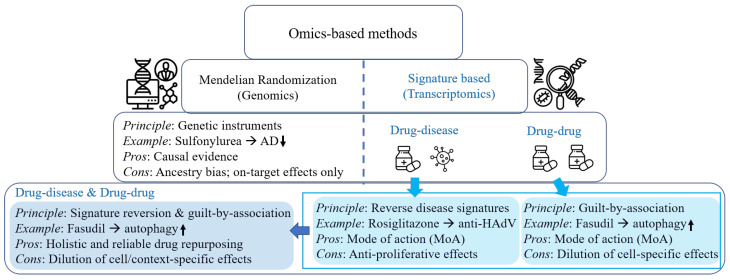
Mendelian randomization (MR) utilizes genomic data to infer causal relationships between drugs and diseases. Signature-based approaches use transcriptomic data for signature reversion or guilt by association, which can be divided into drug-disease and drug-drug approaches.

Omics-based methods provide a systems-level, unbiased view of disease pathophysiology and drug pharmacology by measuring molecular perturbations to infer functional relationships without prior target assumptions [24,73]. These methods are classified into mendelian randomization and signature-based approaches, as illustrated in Figure 3.

Mendelian randomization (MR) uses human genetic variants as natural experiments to infer causal relationships between drug targets and disease risk. An MR study employing genetic proxies for sulfonylurea targets (KCNJ11/ABCC8 genes) demonstrated a causal link between glucose-lowering effects and reduced Alzheimer’s disease risk [20]. While compelling, MR requires strong cis-variants and is often limited to on-target effects within European ancestry populations.

Signature-based approaches using transcriptomic data are divided into drug-disease and drug-drug. The drug-disease approach, Transcriptome Signature Reversion (TSR), identifies compounds that reverse disease signatures to a healthy state. A study comparing human adenovirus infection signatures against LINCS drug profiles via GSEA successfully repurposes rosiglitazone [21]. However, TSR in oncology is confounded by cell viability effects, losing predictive power after correction [22]. Drug-drug approaches focus on pharmacological profile similarity to elucidate the mechanism of action (MoA). Methods like Mode of Action by Network Analysis (MANTRA) and Prototype Ranked Lists (PRLs) cluster drugs into functional communities enriched for shared MoA, enabling “guilt-by-association” predictions [23,85]. This paradigm successfully repurposes Fasudil as an autophagy enhancer and is particularly powerful for understanding system-level pharmacology.

Robust strategies integrate both approaches through a dual matching framework: constructing disease and drug signatures, then cross-referencing reversal hits with functional drug-drug communities to validate mechanistic plausibility [24]. This simultaneously addresses efficacy prediction and MoA elucidation.

The limitations of omics-based methods can be summarized as: (1) data heterogeneity from bulk RNA-seq obscuring tumor heterogeneity; (2) confounding by cell death signals in cancer screens; (3) inability to distinguish driver from passenger events [22]; and (4) population bias in genetic datasets [20]. Future advancements through single-cell profiling, improved correction algorithms, and diverse genomic resources will strengthen omics-based methods.

#### 2.1.3. Fuzzy Logic-Based

Fuzzy logic has emerged as a principled framework for drug repurposing, well suited to biological systems characterized by continuity, ambiguity, and graded relationships. Its appeal lies in bridging the mismatch between discrete computational representations and the inherently non-binary nature of biological phenomena, where drug–target binding, therapeutic response, and adverse effects often vary by degree rather than by absolute state [26]. In this sense, fuzzy logic contributes not only to prediction but also to the formalization of biological uncertainty in computable terms.

Fuzzy logic-based methodologies operationalize this idea through degree-based modeling of uncertain biological information. The fuzzy bipartite local model (FBLM) [25] transforms heterogeneous similarity measures into fuzzy memberships, thereby integrating multiple sources of evidence within a continuous framework. In a SARS-CoV-2 repurposing study [27], genomic ambiguities are represented through fuzzy membership functions and rule-based inference, enabling partial matches to be treated as graded similarities. Similarly, a 2022 cancer study [28] formulated competing therapeutic objectives as fuzzy sets, allowing trade-offs such as efficacy and side effects to be expressed without relying on rigid thresholds or ad hoc weighting. DiffFNN-Med [26] further embeds fuzzy logic into neural architectures, using fuzzy concepts to align model computation with clinical reasoning over imprecise notions such as symptom severity.

Collectively, fuzzy logic provides a theoretically grounded mechanism for representing biological continuity through fuzzification, rule-based inference, and defuzzification. Compared with purely statistical or black-box approaches, it makes uncertainty explicit rather than treating it as residual noise. Its main limitation is the dependence on domain expertise for defining membership functions and rules, yet this also constitutes its strength, since biological ambiguity often requires knowledge-guided formalization. Accordingly, fuzzy methods should be viewed not merely as an algorithmic option but as a modeling paradigm that supports interpretable and continuous reasoning in drug repurposing. Future integration with large language models may help automate rule construction while preserving interpretability.

#### 2.1.4. Adverse Event–Based

Adverse event (AE)–based methods treat spontaneous clinical observations as real-world phenotypic signals for identifying new therapeutic uses of existing drugs [74]. Methodologically, these approaches can be broadly categorized into two groups: methods that rely primarily on AE signals themselves to infer therapeutic potential (AE-primary) and methods that incorporate AE information as an auxiliary evidence layer together with molecular or transcriptomic data (AE-auxiliary).

AE-primary approaches build on the intuition that certain adverse reactions represent phenotypic manifestations opposite to the target disease, and therefore, drugs enriched for such reactions may possess therapeutic potential for that disease. These studies typically begin by mining pharmacovigilance repositories such as FAERS [86] and the WHO pharmacovigilance database [30]. They then apply disproportionality analysis, most commonly the reporting odds ratio (ROR), to quantify statistical associations between drugs and specific AEs. Drugs with significantly elevated reporting frequencies for an inverse phenotype are then prioritized using statistical significance thresholds, ranking metrics, or clinical plausibility filters. One representative example uses hypotension as the phenotypic opposite of hypertension and applies ROR-based disproportionality analysis on FAERS reports, followed by ranking with normalized discounted cumulative gain (NDCG), successfully recovering known antihypertensive drugs while highlighting additional candidates supported by independent clinical evidence [29]. Rather than relying on a single AE term, some approaches construct multidimensional adverse reaction signatures to capture a disease-related pharmacological profile. For instance, a pharmacovigilance study investigating Raynaud’s phenomenon uses erythromelalgia as a vasodilatory inverse phenotype and defines a panel of representative adverse drug reactions (ADRs, such as flushing and vasodilatation) to build ADR signatures; hierarchical clustering of these signatures identified both known vasodilators and novel candidates such as fumaric acid [30]. Similarly, another FAERS-based study targeting hyperhidrosis searches for drugs associated with hypohidrosis or anhidrosis and then applies pragmatic filters such as clinical feasibility, safety considerations, and onset latency to prioritize candidates with rapid and clinically relevant effects [33].

AE-auxiliary approaches integrate AE signals with other biomedical data sources, treating pharmacovigilance information as a complementary layer that strengthens mechanistic plausibility and reduces false positives. In these frameworks, AE-derived signals serve either as an initial filter or as an external validation mechanism for predictions generated from molecular datasets. For example, one psoriasis repurposing study defines “inverse signals” using negative disproportionality thresholds and demonstrates that these AE-derived signals are significantly correlated with reverse gene-expression scores computed from LINCS drug-induced transcriptional signatures [32]. Another integrative pipeline first screens FAERS for drugs associated with reduced reporting of oxaliplatin-induced peripheral neuropathy and then intersects these candidates with compounds affecting disease-relevant genes in the LINCS database. This combined analysis identifies simvastatin as a potential protective agent and subsequently validates its neuroprotective effects in experimental models and retrospective clinical data [31].

AE-based methods leverage real-world patient outcomes for human-centered, scalable hypothesis generation, offering clinical interpretability through signal strength, onset latency, and pharmacologic plausibility, and are enhanced when combined with transcriptomic or gene-network data to reduce false positives and prioritize biologically credible candidates. Consequently, AE-primary methods efficiently narrow candidate pools, and AE-auxiliary methods strengthen confidence for mechanistic and clinical advancement. However, it remains inherently correlational, constrained by spontaneous reporting limitations (under-reporting, bias, confounding, coding inconsistencies, and latency artifacts), necessitating multi-metric assessments (e.g., IC, EBGM [32]), temporal validation, and orthogonal evidence (epidemiology, mechanistic studies, or trials).

### 2.2. Network-Based Methods

**Figure 4:**
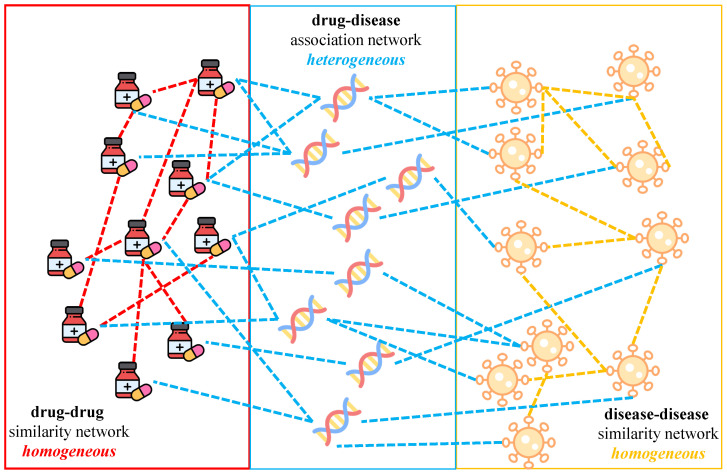
The left side is the drug-drug similarity network, the right side is the disease-disease similarity network. They are connected by common genes to construct a drug-disease heterogeneous network.

Biological networks are commonly used to model interactions among diverse biomedical entities, such as drugs, diseases, genes, and protein targets. Network-based methods exploit these relationships to construct heterogeneous networks and integrate multi-source data, making them a widely adopted strategy in drug repurposing research. A drug-disease heterogeneous network linked by common genes is illustrated in Figure 4. The main advantage of this framework lies in its ability to integrate heterogeneous evidence, which can be further enhanced by LLMs, as discussed in Section 2.3.2.

#### 2.2.1. Graph Mining

Graph mining techniques are widely used in network-based drug repurposing. These methods typically construct networks from drug-disease association matrices and apply graph clustering, random walk, graph diffusion, meta-path, or semantic-based strategies to propagate information and infer missing links, as summarized in Table 2.

##### Graph Clustering

Graph clustering is a fundamental approach in computational drug repurposing, based on the assumption that densely connected modules in biological networks often reflect shared pathways or therapeutic mechanisms. By identifying such modules, these methods infer potential drug-disease associations within the same cluster. The existing studies differ mainly in network design and clustering strategy, and can be broadly grouped into direct clustering, protein complex-mediated approaches, and feature-based clustering.

A representative direct clustering strategy is proposed in [35], where drugs and diseases are connected according to shared genes, with the Jaccard coefficient used to quantify similarity. The resulting heterogeneous network is clustered using Louvain’s modularity [87] and ClusterONE [88], and candidate drug-disease pairs are extracted from the identified modules. This approach provides a global view of the network, but its performance depends strongly on the completeness of gene annotations for both drugs and diseases.

A related strategy incorporates protein complexes as intermediate functional modules [36]. In this study, a tripartite drug–complex–disease network is first constructed, and a drug-disease association network is then derived under biological constraints. Graph clustering is used mainly for validation rather than primary prediction. The resulting drug–disease network is converted into drug-drug and disease-disease similarity networks, which are clustered using ClusterONE [88]. The appearance of coherent clusters containing both predicted and known drugs for a disease provides supporting evidence for the predictions. This framework improves biological plausibility, although it remains challenging to distinguish positive from negative associations automatically.

Other studies combine network-derived features with conventional clustering methods. For example, Ref. [37] first identifies candidate drugs through chemical–chemical and chemical–protein interaction networks, followed by statistical filtering and K-means clustering on feature vectors derived from interaction scores. Candidates that cluster with approved drugs for a disease, such as lung cancer, are prioritized as promising repurposing candidates. This hybrid strategy shows that clustering can be effective for candidate refinement, although it is highly dependent on the quality of the input features.

Overall, graph clustering offers a flexible way to uncover latent therapeutic relationships in biological systems. Its main strength lies in integrating heterogeneous evidence at the systems level. However, these methods often depend on data completeness, may be difficult to interpret biologically, and generally cannot determine the therapeutic direction of an association.

##### Random Walk

The application of network-based methods provides powerful computational tools for drug repurposing, among which random walk algorithms stand out for their ability to infer novel drug-disease associations by leveraging the global topology of biological networks. The core premise involves modeling biological entities (e.g., drugs, diseases, genes and miRNAs) as nodes in a network, with edges representing relationships between them. A random walk simulation starts from known “seed” nodes (e.g., drugs that treat a specific disease) and traverses the network. The probability of the walker reaching any particular node after many steps reflects its association strength with the seed set, enabling the identification of potential new therapeutic indications.

The evolution of random walk methodologies reflects a trend towards increasing sophistication in network construction and propagation dynamics. The MBiRW [38] framework, an early pioneer, emphasizes the critical role of similarity network refinement. Its innovation begins with enhancing drug and disease similarity matrices through logistic transformation and topology-aware clustering before executing a bidirectional random walk on the resulting heterogeneous network. A key insight is the introduction of two distinct parameters to control the maximum steps for the drug and disease walks separately, acknowledging their potentially different topological characteristics. Shortly after, the TP-NRWRH (Two-Pass Network-based Random Walk with Restart on Heterogeneous Networks) [39] method demonstrates the advantage of incorporating dual perspectives. Instead of a single walk, TP-NRWRH performs two separate random walks with restart (RWR): one drug-centric and one disease-centric. The final association score is derived by averaging the results from these two passes, a strategy that comprehensively captures network proximity from both viewpoints and set a high benchmark for prediction accuracy. To address the limitation of fixed propagation parameters, the DR-IBRW (Drug Repositioning based on Individual Bi-Random Walks) [40] model introduces the concept of node-specific walk lengths. Arguing that each node in the network should have a unique level of topological influence, DR-IBRW calculates individualized walk lengths for each drug and disease node. This adaptive propagation strategy allows the algorithm to account for structural heterogeneity more effectively than methods using a fixed walk-length. Beyond the typical drug-disease network, the random walk framework has been successfully applied to other biological relationship networks. For instance, a study [41] constructs a drug–drug interaction network based on shared miRNA regulation. A standard RWR algorithm is then applied on this homogeneous network, using known drugs for a disease as seeds to rank candidate drugs. This approach underscores the flexibility of the random walk paradigm to integrate diverse data types, such as miRNA-mediated mechanisms, for drug repurposing.

When comparing these methods, a clear evolution in design is apparent. MBiRW’s primary contribution lies in its rigorous pre-processing of similarity data. In contrast, TP-NRWRH’s strength is its symmetric two-pass propagation, while DR-IBRW offers a more nuanced, topology-aware walking strategy. The miRNA-based approach exemplifies the model’s flexibility in adapting to different network structures. The overarching advantages of random walk approaches are their ability to exploit the global structure of biological networks and their proven high performance in computational validation. However, the field must also contend with several inherent limitations. The predictive accuracy of these models is highly dependent on the quality and completeness of the underlying data, such as similarity metrics and known associations. Furthermore, parameters like the restart probability require careful tuning, and the algorithms can function as black boxes, making the biological interpretation of specific predictions non-trivial.

In summary, random walk algorithms have established themselves as a cornerstone of network-based drug repurposing. The methodological progression from basic bidirectional walks to multi-perspective and individualized propagation walk lengths highlights a continuous effort to enhance biological relevance and predictive power. Future research will likely focus on integrating ever-more-diverse data types into heterogeneous networks and developing more efficient, interpretable, and scalable random walk variants to uncover novel therapeutic indications hidden within the interconnected landscape of biological data.

##### Graph Diffusion

Graph diffusion has been widely adopted in computational drug repurposing to address the fundamental challenge of association sparsity in biological networks [89]. These methods are grounded in the principle that entities connected through multiple paths in a graph are functionally related, making diffusion ideal for inferring latent interactions between drugs and diseases [90]. Originally applied in social networks and web ranking (e.g., the PageRank algorithm [91]), graph diffusion techniques can be adapted to biological link prediction by simulating information propagation across heterogeneous networks, effectively leveraging high-order connectivity [92,93].

The application of these techniques evolves along a broad methodological spectrum, progressing from non-parametric approaches to embedding learning integration and more recently to hard negative sampling. Among non-parametric approaches, BGMSDDA [42] employs bipartite graph diffusion directly as its prediction mechanism. It first densifies the sparse drug-disease adjacency matrix using a weighted K-nearest neighbors algorithm (WKNKN) and integrates multiple drug and disease similarities. These are used to construct diffusion matrices that propagate association probabilities through iterative resource allocation, entirely without learnable parameters. In contrast, later works integrate graph diffusion into embedding learning frameworks to enhance representation learning. For instance, HGCL-DR [43] uses Personalized PageRank-based graph diffusion (PPR-GDC) [94] not for prediction but as a feature-smoothing operator to capture long-range dependencies in drug–drug and disease–disease homogeneous networks. These diffused features are then fed into graph convolutional networks and contrastive learning modules to learn robust node embeddings for downstream classification. A more recent innovation uses diffusion models for generating hard negative samples. GDNDGP [44] (Preprint) leverages a graph diffusion network to generate hard negative gene embeddings for contrastive learning. By diffusing and denoising gene embeddings conditioned on drug embeddings, it creates challenging negative samples that improve the discrimination of drug–gene association classifiers.

Collectively, these methods demonstrate the versatility of graph diffusion, albeit with distinct trade-offs. The non-parametric approach (e.g., BGMSDDA) is computationally efficient and interpretable but relies heavily on hand-crafted similarities and damping factors. The approach that integrates diffusion into embedding learning (e.g., HGCL-DR) gains representational power at the cost of increased complexity and reduced interpretability. The hard negative sampling approach (e.g., GDNDGP) offers a novel solution to specific learning challenges but introduces additional training overhead. Future research will likely focus on scaling these methods to larger heterogeneous networks, improving their interpretability, and further exploring conditional diffusion models.

##### Meta-Path

Meta-paths are originally developed for mining heterogeneous information networks (HINs), which contain multiple node and relation types. In these networks, a meta-path defines a composite relation between two entity types through a sequence of node and edge types, and it has been widely used in social network analysis and recommendation systems [95,96]. This framework is particularly suitable for capturing semantic relationships and similarity in complex networks.

In computational biology, meta-paths naturally fit the drug repurposing problem because biomedical data, including drugs, proteins, diseases, side effects, and functional annotations, can be represented as a heterogeneous network. Drug–disease prediction can, therefore, be formulated as a link prediction task over biologically meaningful paths, such as Drug → Protein → Disease or Drug → Gene Ontology → Disease. Such paths can reflect shared mechanisms, functional similarity, or topological proximity, providing both interpretability and a principled way to integrate heterogeneous data.

Meta-path-based methods generally follow a common pipeline: constructing a heterogeneous network, defining biologically meaningful meta-paths, generating path-based feature matrices for drug-disease pairs, and applying machine learning models for prediction. EMP-SVD [45] relies on manually defined meta-paths and uses singular value decomposition together with ensemble classifiers. This idea is extended by incorporating Gene Ontology into meta-path-based gene ontology profiles (MGP-DDA) [46], which improved biological interpretability. More recent methods, such as HSGCLRDA [47], use graph attention networks to aggregate information within and across meta-paths while also introducing hierarchical negative sampling and contrastive learning to improve representation quality and robustness.

The main advantage of meta-path methods is their interpretability, since the selected paths often correspond to plausible biological mechanisms. They also provide an effective way to integrate multiple data sources in a unified framework. However, a key limitation is the dependence on manually predefined meta-paths, although attention-based and learnable mechanisms can partially alleviate this issue. In addition, enumerating path instances can be computationally expensive in large networks.

In summary, meta-path methods have evolved from handcrafted path engineering and traditional machine learning to more adaptive graph representation learning models. Future research is likely to focus on self-supervised learning and scalable meta-path reasoning for drug repurposing.

##### Semantic-Based

Semantic-based methods extend network exploration from topological correlation to biologically interpretable hypotheses by focusing on semantic subgraphs with explicit biological meaning. In general, these methods construct semantic networks from large-scale biochemical databases and then mine semantic paths or subgraphs to predict novel drug-disease associations [2]. Their development has been closely tied to the integration of biomedical knowledge graphs.

Early methods established the statistical basis of semantic network mining. SLAP predicts missing links by combining topological and semantic scores and then transforms pattern-based scores into z-scores to distinguish functionally similar compounds from structurally similar ones [50]. SemEP further introduces an unsupervised edge-partitioning strategy that allows drugs and targets to belong to multiple clusters, thereby accommodating polypharmacology while maximizing cluster density under semantic constraints [51].

A further step comes from formal knowledge representation. Semantic Web technologies have been used in breast cancer repurposing to represent relationships among drugs, genes, diseases, SNPs, and pathways through OWL, enabling automated reasoning to identify new indications, such as the potential link between tamoxifen and ovarian cancer [52]. To improve scalability, DReSMin introduces semantic graph pruning and subgraph splitting, achieving near-linear scalability for large heterogeneous networks while also defining a MeSH-based semantic distance metric to distinguish therapeutic indications from adverse effects [53,97]. More recently, DRGCL integrates graph contrastive learning to align topological and semantic views through separate encoders, thereby improving predictive performance through consistent cross-view representations [49].

In general, semantic-based methods provide three main advantages over conventional network approaches. First, they produce biologically interpretable predictions, since semantic subgraphs often correspond to meaningful molecular or clinical mechanisms. Second, formal ontologies and logical reasoning can capture complex relationships that may be missed by purely statistical methods. Third, semantic frameworks can integrate diverse data types, ranging from chemical structures to clinical information, within a unified representation. Despite these strengths, several challenges remain, including dependence on the completeness and consistency of ontologies and databases, high computational cost for large-scale knowledge graphs, and limited flexibility in manually defined semantic rules or meta-paths.

#### 2.2.2. Matrix Factorization or Matrix Completion

Drug repurposing can be formulated as a recommendation problem, where drugs are treated as “users”, diseases as “items”, and known drug-disease associations as observed preferences. The task is to infer unknown associations from a sparse interaction matrix, which has led to two major classes of methods, as summarized in Table 3.

Matrix factorization (MF) methods decompose the sparse drug-disease association matrix *R* into lower-dimensional latent representations, typically a drug-feature matrix *U* and a disease-feature matrix *V*, such that $R\approx UV^{T}$. Early methods such as Similarity Constrained Matrix Factorization (SCMFDD) [55] incorporate drug–drug and disease–disease similarities as biological constraints. Later methods, including DisDrugPred [56] and Multi-Similarities Bilinear Matrix Factorization (MSBMF) [59], further improve robustness by integrating multiple similarity sources. Extending the same idea beyond matrices, Nonnegative Tensor Decomposition for Drug Repositioning (NTD-DR) [60] models the problem as tensor factorization. Despite their flexibility, MF methods remain limited by the cold-start problem since they cannot directly infer associations for novel drugs or diseases that are absent from the training matrix.

In contrast, matrix completion (MC) methods infer missing entries by assuming that the full drug-disease association matrix is low-rank. Drug Repositioning Recommendation System (DRRS) [54] performs completion through nuclear norm minimization. Overlap Matrix Completion (OMC) [57] improves scalability by leveraging bilayer network completion. To better handle novel drugs or diseases, inductive strategies are also introduced. For example, DRIMC [58] combines weighted K-nearest neighbor preprocessing with Bayesian inductive matrix completion, thereby smoothing association information and improving generalization to unseen cases.

Overall, MF and MC represent two closely related yet distinct strategies: MF emphasizes latent feature learning and interpretability, whereas MC focuses on directly recovering missing associations under low-rank assumptions. Bayesian inductive matrix completion further bridges prediction accuracy and generalization capability, reflecting the gradual maturation of matrix-based drug repurposing methods.

Matrix-based drug repurposing has, therefore, evolved from basic MF and MC models to hybrid frameworks that integrate multi-source data, mitigate cold-start issues, and improve robustness through more advanced regularization and inductive learning strategies. This development highlights the continuing balance among interpretability, predictive performance, and practical applicability.

### 2.3. Data-Driven

#### 2.3.1. Text Mining-Based

**Figure 5:**
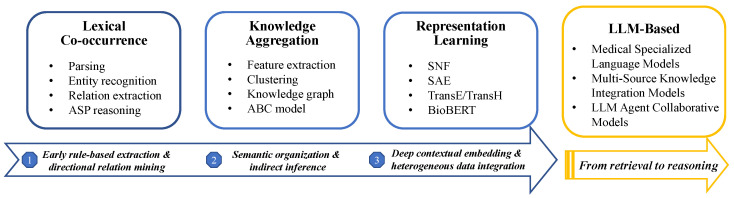
The evolutionary trajectory of text mining methodologies in drug repurposing. The timeline illustrates the transition from early lexical co-occurrence and rule-based reasoning to contemporary knowledge aggregation and representation learning, culminating in the integration of large language models (LLMs) for advanced reasoning and hypothesis generation.

Text mining serves as a pivotal computational strategy for drug repurposing by systematically extracting structured information from vast biomedical literature and databases. It transforms textual data into actionable knowledge to infer drug–disease interactions, offering scalability to analyze millions of publications, integration of diverse data types (mechanisms, indications, adverse effects), and detection of both direct and indirect relationships beyond manual curation. The development of these methods is illustrated in Figure 5.

The field has evolved from semantic relationship extraction to advanced machine learning integration. Early foundational work [64] establishes a framework using syntactic and semantic parsing to extract directional interactions, formally encoded with Answer Set Programming (ASP) [98,99] for automated logical reasoning and mechanistic inference of novel indications. Subsequent methods explore complementary strategies: DrugQuest [61] employs feature extraction and clustering to represent drugs as binary vectors, enabling repurposing candidate discovery through semantic similarity; BEST [62] functions as an entity-centric search engine for rapid literature-based candidate identification; a Parkinson’s disease study [66] constructs a disease-specific knowledge graph and applies representation learning models (TransE [100], TransH [101]) for prediction; and a comprehensive pipeline [65] combines relationship extraction with the ABC model [102] for indirect interaction inference, introducing a ranking system based on drug vector similarity.

Recent advances integrate text mining with similarity networks and deep learning. HeTDR [63] exemplifies this by processing nine drug-related networks via positive pointwise mutual information (PPMI) matrices, fusing them with similarity network fusion (SNF) [103] and distilling features using a sparse autoencoder (SAE) [104]. For disease features, it employs BioBERT [75] to capture contextualized semantics, culminating in embedding-based prediction that preserves network topology and attribute proximity. This trajectory reveals progressive sophistication: relationship extraction evolves from syntactic parsing to deep contextual representations; inference mechanisms advance from logical reasoning to embedding-based prediction; and knowledge representation shifts from logic facts to low-dimensional embeddings, enabling capture of complex semantics and seamless integration with heterogeneous biological data.

In summary, text mining provides versatile tools for drug repurposing, offering mechanistic hypothesis generation, intuitive exploration, rapid retrieval, and integration with predictive models. Challenges include potential source biases, dependency on entity recognition quality, and the computational complexity of advanced frameworks. Notably, HeTDR’s BioBERT implementation [63] signals a paradigm shift from custom NLP pipelines to pre-trained biomedical language models, foreshadowing the use of large language models (LLMs) as core engines for reasoning and hypothesis generation, a topic explored in Section 2.3.2.

#### 2.3.2. Large Language Model-Based

Compared with traditional drug development, drug repurposing offers advantages in terms of lower costs and shorter timelines, yet it heavily relies on manual screening and limited data integration. To date, the well-known examples—including early applications of minoxidil for hair loss [105] and sildenafil for erectile dysfunction [106], as well as the more recent use of aspirin for colorectal cancer [107]—have primarily stemmed from a deep understanding of drug pharmacology and retrospective analyses of clinical effects when drugs are prescribed for their original indications [4]. Although biological mechanism-driven, network-based, and text mining-based methods have advanced the field, their limitations become evident when confronted with increasingly abundant heterogeneous biomedical data and the complex biological processes underlying drug repurposing.

In recent years, large language models (LLMs) have demonstrated growing capabilities in contextual semantic understanding, unified representation learning, and zero-shot task adaptation [108], driving transformation across various industries [109,110]. Their broad learning and knowledge representation abilities in handling complex and diverse tasks offer potential novel methodologies for drug repurposing research.

It is important to note that LLMs do not represent a discontinuous breakthrough emerging in isolation; rather, they constitute a natural progression and deepening of earlier data-driven paradigms. As discussed in Section 2.3.1, text mining-based methods have already achieved preliminary success in extracting structured knowledge from unstructured literature through techniques such as lexical co-occurrence analysis and knowledge graph construction. The advent of LLMs fundamentally represents a scaling-up and capability leap within this paradigm: they learn statistical patterns that can approximate some of the core tasks of text mining, information extraction, relation recognition, and knowledge aggregation as part of the pre-training process. However, this statistical learning does not guarantee reliable structured biomedical knowledge extraction without rigorous validation; LLMs do not “understand” biology in a causal sense, and their outputs must be treated as hypotheses rather than facts. In other words, LLMs can be understood as large-scale, end-to-end enhanced text mining engines, but with the important caveat that their outputs require orthogonal verification.

**Figure 6:**
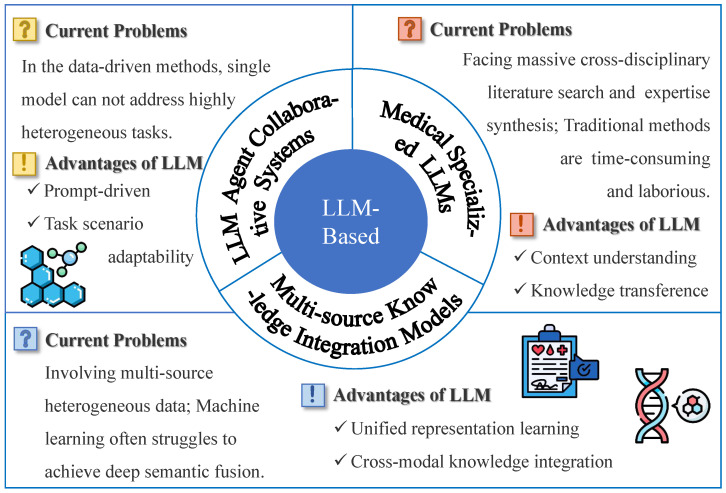
A conceptual generalization of large language model-based methods for drug repurposing. This figure provides an overview of three methods for drug repurposing using large language models (LLMs). Specifically, in each category of research, we first describe the limitations of traditional artificial intelligence techniques and then list the unique advantages of large language models in overcoming these limitations.

This section begins by analyzing the challenges inherent in drug repurposing predictions based on previous methods. As illustrated in Figure 6, we synthesize the characteristics and strengths of large language models to summarize and organize current research on LLM-based drug repurposing, with a focus on how LLMs may inject new momentum into the field. Nevertheless, it must be recognized that current LLM-based applications in drug repurposing remain at an early stage and have several fundamental limitations, which will be discussed in detail in Section 2.3.3.

##### Medical Specialized Language Models

Drug repurposing requires extensive cross-disciplinary knowledge spanning molecular biology, pharmacology, and bioinformatics, often necessitating comprehensive retrieval and analysis of vast biomedical literature [67] (Preprint). Despite their pivotal role in systematically extracting information from unstructured texts, previous text mining-based methods exhibit inherent limitations, including a reliance on the quality of entity recognition and relationship extraction, and challenges in capturing deep semantic and contextual information.

Medical specialized large language models, fine-tuned or pre-trained on large-scale biomedical corpora such as PubMed papers and clinical guidelines, are better suited for understanding biomedical terminology and complex relationships [67] (Preprint) and [68]. These models have demonstrated preliminary capabilities in named entity recognition, relation extraction, and question answering within the biomedical domain. For drug repurposing, medical specialized LLMs can be employed to assist in extracting drug-disease associations, mechanisms of action, and potential side effects, potentially reducing manual curation efforts. However, these capabilities have mostly been evaluated on benchmark datasets rather than in prospective drug repurposing pipelines, and their generalizability to novel indications remains to be established.

A representative example is Y-Mol [67] (Preprint), a multiscale biomedical knowledge-guided LLM for drug development. Y-Mol integrates biomedical knowledge graphs, literature, and molecular structures to enable tasks such as drug–target interaction prediction and drug repurposing candidate screening. The authors report promising results on retrospective tasks, but independent replication and prospective validation are lacking.

Similarly, a study leveraging generative AI [68] prioritizes drug repurposing candidates for Alzheimer’s disease by prompting GPT-4 with structured queries derived from biomedical databases, followed by real-world clinical validation using electronic health records. This study suggests that LLM-generated hypotheses, when combined with real-world data, identify promising candidates such as anti-hypertensive drugs for Alzheimer’s disease. Notably, this study has undergone peer review and includes clinical validation. Nevertheless, its findings require replication in independent cohorts.

Despite these advances, medical specialized LLMs still face challenges related to data bias (e.g., over-representation of certain diseases or populations), hallucinated outputs, and the need for rigorous validation pipelines. Moreover, their performance heavily depends on the quality and coverage of the training corpora, and they may not generalize well to emerging diseases or understudied indications. At present, medical specialized LLMs should be viewed as assistive tools for hypothesis generation rather than standalone decision-making systems.

##### Multi-Source Knowledge Integration Models

Drug repurposing inherently requires the integration of heterogeneous data sources, including genomic, transcriptomic, proteomic, chemical, clinical, and literature data. Traditional approaches often combine these sources through simple concatenation, similarity fusion, or ensemble learning, but they struggle to achieve deep semantic alignment across different modalities. For instance, integrating molecular structures (graph data) with textual descriptions or knowledge graph triples remains challenging due to the disparate representations and semantic gaps across modalities. Existing methods typically handle each modality separately and then combine predictions or features, which lacks a general and seamless framework for multi-modal fusion. Consequently, existing methods remain inadequate to achieve deep and semantically consistent integration across multi-modal sources.

Multi-modal large models [111] (Preprint) and [112] have been explored for their unified representation learning and cross-modal knowledge integration capabilities, enabling the consolidation of structured and unstructured information, such as texts, knowledge graphs, and molecular structures, and thereby achieving joint modeling of diverse data sources. Unified representation learning refers to the ability of LLMs to map different modalities of data from various sources (e.g., text, images, graph structures, sequences) into the same semantic space, learning shared latent semantic representations. This characteristic of aligning multi-source data allows LLMs to facilitate joint modeling of disparate data sources, enabling mutually complementary learning across modalities and enhancing and enriching data representation capabilities.

Researchers have recognized the advantages of LLMs in unified representation learning and cross-modal knowledge integration and have combined them with methods such as graph neural networks, molecular graphs, structural data, protein interaction networks, and knowledge graphs to propose multi-source knowledge integration solutions tailored for drug repurposing. However, the extent to which current LLMs achieve true semantic alignment for biomedical modalities remains an open research question, and most applications still rely on relatively shallow fusion techniques.

Utilizing large language models (LLMs) to mine biological databases represents a novel approach for identifying drug repurposing candidates for Alzheimer’s disease (AD) [70]. A knowledge graph database is constructed by integrating AD-related biological processes from Gene Ontology and drug information from DrugBank. On the basis of that, using a locally deployed Llama3 model, the repurposing potential of drugs is evaluated via zero-shot prompting. Based on the regulatory effects of drugs on AD-associated biological processes, the model generates a score between 0 and 1 along with explanatory reasoning. Among the evaluated drugs, several high-potential repurposing candidates are identified and subsequently undergo manual review, hallucination detection, and adverse effect evaluation. The study further correlates model outputs with geographical distributions of existing clinical trials, highlighting that the results may be influenced by regional biases inherent in the training data.

Large language models (LLMs) may support graph-structured search and relational reasoning tasks. Employing LLMs as knowledge extraction tools may enhance the performance of graph neural networks (GNNs) in the prediction of drug-disease associations [69]. This approach leverages GPT-4 for zero-shot prompting to generate high-quality biomedical descriptions of drugs and diseases, which are subsequently transformed into numerical embeddings. Three distinct architectures—node feature fusion, dual-channel GNN, and GNN-autoencoder—are designed to explore optimal integration strategies between LLM-derived embeddings and heterogeneous graph topological features. The authors report improved AUROC and AUPRC compared to GNN baselines, suggesting that LLM-generated semantic features can complement structural graph information. Nevertheless, the added value of LLMs over simpler text embedding methods is not systematically compared, and the computational cost of API-based LLM inference is substantial.

**Figure 7:**
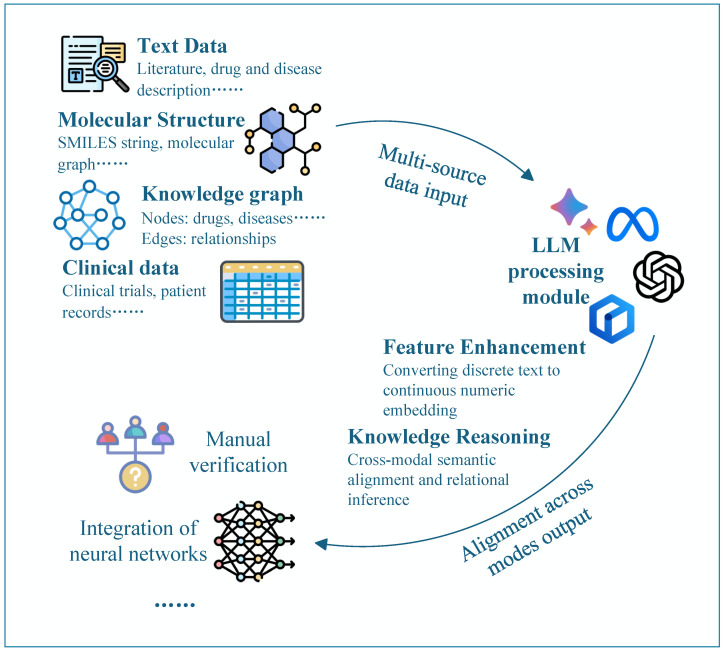
Implementation process of multi-source knowledge integration models. LLMs serve as central engine for cross-modal alignment, enabling the semantic-level integration of heterogeneous data through multiple roles.

In multi-source knowledge integration models, as shown in Figure 7, large language models (LLMs) may serve as a central engine for cross-modal alignment, functioning both as feature augmenters that create contextualized embeddings from text and as knowledge-enhanced reasoners that infer over structured biological knowledge. This dual capacity unifies structured and unstructured context, enabling a flexible fusion of heterogeneous data. However, achieving genuine semantic-level integration beyond superficial feature concatenation remains challenging, and most current implementations still rely on post hoc combination rather than end-to-end joint modeling.

##### LLM Agent Collaborative Models

A typical drug-repurposing workflow often includes hypothesis generation, preclinical evaluation, and clinical validation [4]. Artificial intelligence plays a significant role in the first stage of computation and hypothesis generation. In data-driven methods, the first stage relies entirely on big data and computational analysis. AI technology may assist in completing subtasks such as multi-source data integration, potential association information extraction, drug–disease pathway prediction, and molecular docking simulations, thereby advancing drug repurposing efforts. Due to the high heterogeneity of subtasks, traditional single models face certain limitations when handling multi-dimensional, highly complex tasks.

Prompt-driven approaches, as a core concept in LLM applications, serve as a task control language and behavior-scheduling mechanism. Using prompt technology, LLMs can execute instructions under different roles based on system prompts or contextual information, improving their adaptability and generalization capabilities across various task scenarios [113]. Furthermore, since LLMs are trained on extensive data from diverse domains, they can still provide reasonable responses to tasks not explicitly encountered during training by understanding task descriptions and analogizing known knowledge.

Even before the concept of LLMs emerges, agents are constructed using rule matching [114], reinforcement learning [115], and other methods are already applied in academia and industry for auxiliary decision-making in complex environments. When LLMs demonstrate powerful language understanding, generation capabilities, and broad generalizability, the idea of using LLMs as the brain of intelligent agents is proposed [116,117]. Compared to traditional agents, LLM agents can handle more complex and diverse tasks with greater flexibility and adaptability. Through the construction of multiple agent roles to simulate expert teams, complex tasks can be broken down meticulously to directionally solve specialized problems. Leveraging their role-playing capabilities, LLM agents can bridge the gap between general AI capabilities and the nuanced requirements of complex research, improving the consistency and reliability of outcomes. They are widely used in software development [118,119], economics [117], social sciences [120,121], and other fields.

In the pharmaceutical domain, a growing number of scholars have noticed this new advancement. Using prompt technology to set role-playing and task boundaries for LLMs, they address complex specialized problems through multi-role collaboration.

DrugAgent [72] is a multi-agent cooperative reasoning system based on LLMs for drug–target interaction prediction. It innovatively organizes multiple LLM agents into a team with specialized roles and leverages the Chain-of-Thought (CoT) and ReAct frameworks to integrate multi-source evidence. By enabling interactive reasoning among LLM agents, the system can enhance both prediction accuracy and interpretability in controlled retrospective experiments. However, the reported results are based on standard benchmark datasets, and prospective validation or real-world deployment has not been demonstrated.

An LLM-based multi-agent automated collaboration framework can be designed to address tasks in the drug discovery process, such as ADMET prediction, high-throughput screening, and drug–target interaction prediction [71]. The framework first employs an LLM planner to develop high-level solutions, followed by an LLM instructor that integrates domain knowledge and specialized tools to ensure plan execution. This enables a low-threshold, highly reliable automated workflow spanning from data preprocessing to model optimization. While conceptually compelling, the framework has only been tested on a limited set of tasks, and its generalizability to diverse drug repurposing scenarios remains to be established.

**Figure 8:**
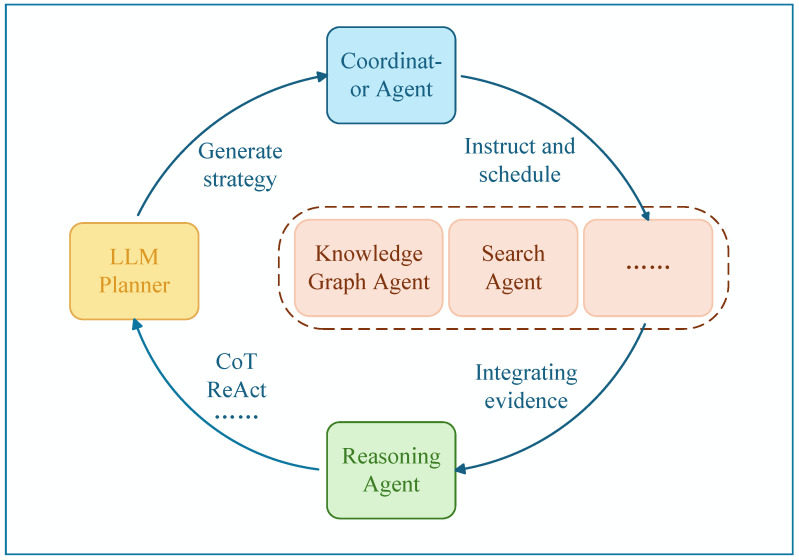
Implementation process of LLM agent collaborative models. Agents simulate expert teams via structured prompting (Chain-of-Thought, ReAct), enhancing predictive accuracy, interpretability, and reliability for automated hypothesis generation in AI-driven drug repurposing.

In summary, LLM agent collaborative models introduce a transformative multi-agent paradigm for computational drug repurposing, potentially addressing the limitations of single-model systems in handling highly heterogeneous and multi-stage tasks. These LLM-based agent systems organize specialized roles for distinct subtasks, as demonstrated by frameworks that conduct evidence-based reasoning for drug–target interaction prediction and deploy automated workflows that decompose complex problems into manageable, role-specific operations. Figure 8 illustrates the construction approach of these LLM agent collaborative models. By simulating expert teams and utilizing structured prompt frameworks such as Chain-of-Thought (CoT) and ReAct, these systems can enhance prediction accuracy. Moreover, these approaches also achieve certain improvements in terms of enhancing the interpretability and automation of the hypothesis generation process. Nevertheless, the field is nascent, and rigorous comparative studies against non-agent baselines, as well as prospective experimental validation, are needed before these methods can be recommended for routine use in drug repurposing.

#### 2.3.3. Hallucinations in Large Language Models: Risks and Impacts on Drug Repurposing

Despite the substantial advancements enabled by LLM-based methods, their application in drug repurposing is critically challenged by hallucination—the generation of outputs that are fluent and coherent yet factually incorrect, unsupported by available evidence, or inconsistent with input context [122,123]. Accumulating theoretical and empirical evidence indicates that hallucination is not merely an engineering flaw but a structural property of autoregressive language models, which are optimized for likelihood maximization of token sequences rather than for factual veracity. Consequently, no existing LLM can guarantee zero hallucination under the current architectural and training paradigms. This intrinsic limitation is particularly consequential in drug repurposing, where erroneous outputs can propagate through downstream analyses, misdirect experimental efforts, and—when translated to clinical contexts—potentially compromise patient safety. Because general taxonomies of hallucination causes have been extensively reviewed elsewhere [123], this section focuses on drug-repurposing-specific manifestations, impacts, and mitigation strategies.

##### Concrete Hallucination Risks in Drug Repurposing

Four types of hallucinations pose direct threats to research integrity and translational validity. First, fabricated drug–target relationships occur when a model asserts a direct interaction between a compound and a protein without supporting evidence, leading researchers to invest resources in biologically implausible targets. Second, unsupported disease mechanisms involve the generation of plausible-sounding but factually incorrect causal narratives, such as inventing a novel signaling pathway that has never been experimentally observed, thereby distorting mechanistic understanding. Third, invalid citation generation refers to the production of non-existent or misattributed references, including fabricated PubMed identifiers, author names, or journal citations, which undermines traceability and verification. Fourth, biased clinical trial evidence arises from skewed training data, leading to predictions that are not generalizable or that neglect rare but clinically significant adverse effects.

##### Propagation of Hallucinations Through the Drug Repurposing Pipeline

Hallucinations do not remain as isolated factual errors; they affect multiple decision points. In terms of biomedical inference, a fabricated claim about a drug’s mechanism of action can misdirect hypothesis testing, causing researchers to design irrelevant assays or pursue false combination strategies. Regarding mechanistic interpretation, LLMs often generate confident but ungrounded explanations, which clinicians or biologists may mistakenly treat as genuine insights, hindering validation efforts. In candidate prioritization, a hallucinated high-confidence score for an invalid target can divert resources away from genuinely promising candidates. The associated risk is asymmetric: over-trusting a confident but incorrect prediction is generally more dangerous than conservatively discarding a true positive—a finding consistent with analyses of medical AI where “almost correct” errors pose the greatest hazard [124].

##### System-Level Barriers to Reliable Deployment

Several practical constraints hinder the use of LLMs in drug repurposing beyond the hallucination content itself. Reproducibility is compromised by high sensitivity to minor variations in prompts, decoding parameters, and model versions. Benchmark scarcity reflects the absence of standardized benchmarks for evaluating hallucinations specifically in target–disease association inference or mechanism generation; existing medical QA benchmarks do not capture the complexity and domain specificity of drug repurposing workflows. Regulatory concerns arise because agencies such as the FDA and EMA currently lack established frameworks for validating AI-generated therapeutic hypotheses; unverified LLM outputs cannot be accepted as evidence in regulatory submissions without rigorous orthogonal validation, including experimental or clinical confirmation. Finally, a lack of experimental validation pipelines persists, as the majority of LLM-based drug repurposing studies conclude at the computational prediction stage, leaving the real-world utility and safety of LLM-generated candidates largely unproven.

##### Mitigation Strategies and Limitations

Several techniques have been proposed to reduce hallucination rates, including retrieval-augmented generation (RAG), self-reflection, multi-agent cross-validation, and decoding-time memory control. While these methods can lower the frequency of hallucinations, none eliminate them entirely. RAG grounds outputs in external knowledge bases but may fail when retrieved documents themselves contain inaccuracies or when the model disregards retrieved evidence. Self-reflection can correct some errors but has been shown to degrade performance on already-correct answers. Multi-agent debate improves factuality at a substantial computational cost and does not guarantee correctness in novel or ambiguous cases. Decoding-time memory control modifies generation mechanisms but introduces additional latency.

Given these limitations, a pragmatic consensus is emerging in the literature: any LLM-generated hypothesis intended for drug repurposing must be treated as a candidate requiring orthogonal validation. Such validation may include computational cross-checking (e.g., network propagation, molecular docking, independent literature verification) and, for high-priority candidates, experimental assays such as binding studies, cell-based models, or animal efficacy tests. Integrating LLMs as hypothesis generators within a broader human–AI co-validation framework—rather than as autonomous decision-makers—offers a realistic path toward safe and effective use in drug repurposing.

### 2.4. Cross-Method Comparison

Taken together, the computational paradigms reviewed in this article differ not only in algorithmic form but also in their data dependence, inferential assumptions, and validation requirements. Within the biological mechanism-driven category, structure-based methods are most appropriate when reliable target structures are available and the primary objective is to obtain atomic-level mechanistic insight through molecular docking and molecular dynamics simulations. Omics-based methods are better suited to causal inference and signature reversal analysis, as they leverage genomic or transcriptomic perturbation data to infer drug–disease relationships or mechanisms of action. Fuzzy logic-based methods formalize biological ambiguity through fuzzification, rule-based inference, and defuzzification, thereby offering interpretable modeling of graded biological phenomena, although they depend heavily on expert-defined membership functions and rules. Adverse event-based methods exploit real-world pharmacovigilance signals to generate clinically grounded hypotheses, but their outputs are constrained by spontaneous reporting bias, confounding, and the need for orthogonal validation.

In the network-based category, graph mining and matrix factorization/completion are especially effective for large-scale candidate prioritization because they integrate heterogeneous evidence at the systems level and infer latent drug–disease associations from sparse interaction networks. Graph mining methods, including graph clustering, random walk, graph diffusion, meta-path, and semantic-based strategies, are generally well suited to diseases with sufficient network connectivity and prior biological annotations. Matrix factorization and completion methods are useful for reconstructing missing associations in sparse matrices, but their performance is strongly influenced by cold-start settings, similarity construction, and negative sampling strategies. Compared with biological mechanism-driven methods, network-based methods typically provide broader coverage and stronger ranking capability, but their predictions are often less directly interpretable and more vulnerable to data leakage or overly optimistic retrospective evaluation.

The data-driven category, which includes text mining-based and LLM-based methods, extends drug repurposing from curated biomedical relations to unstructured textual evidence and cross-source knowledge integration. Text mining-based methods are valuable for literature-based candidate identification, indirect relationship inference, and knowledge graph construction, whereas LLM-based methods offer superior contextual understanding, task flexibility, and multi-source semantic integration. However, both classes remain sensitive to source bias, entity recognition errors, incomplete evidence, and, in the case of LLMs, hallucination and reproducibility concerns. Accordingly, data-driven methods are best viewed as hypothesis generation and evidence synthesis tools rather than stand-alone validators.

Overall, no single paradigm is universally superior. Biological mechanism-driven methods are most suitable for mechanistic interpretation and experimental confirmation; network-based methods are most effective for large-scale prioritization and association mining; and data-driven methods are particularly powerful for knowledge discovery and semantic integration. In practice, the most robust drug repurposing workflows often combine these paradigms in a staged manner, for example by using network-based or data-driven methods to generate candidates and then applying biological mechanism-driven methods for validation and explanation. Therefore, the critical issue is not which paradigm replaces the others but how complementary methods can be integrated to balance coverage, interpretability, scalability, and translational reliability.

## 3. Data Sources, Evaluation Metrics and Validation Strategies

Across drug repurposing studies, the choice of data sources, evaluation metrics, and validation strategies varies substantially across methodological paradigms. As a result, direct performance comparison between studies is often not straightforward because different datasets, label constructions, and train-test splits can lead to markedly different outcomes. In addition, most current studies rely primarily on retrospective validation against known drug–disease associations, whereas only a limited number of works extend validation to in vitro or in vivo experiments, or to clinically relevant evidence.

### 3.1. Biological Mechanism-Driven

#### 3.1.1. Structure-Based

Structure-based methods commonly integrate structural, chemical, and biological resources to support docking, molecular dynamics (MD), and related simulations. Protein structures are typically obtained from the Protein Data Bank (PDB) [125], while drug structures and annotations are retrieved from databases such as DrugBank, PubChem, and ChEMBL [126,127,128]. Disease-related genes, pathways, and functional annotations are often incorporated from OMIM, gene ontology (GO), protein–protein interaction resources, and literature-derived datasets, enabling a more comprehensive representation of the biological context [129,130,131].

The evaluation in structure-based studies usually focuses on binding affinity and complex stability. For molecular docking, commonly reported measures include docking score, binding energy, pharmacophore fit, and pose deviation metrics such as RMSD. For MD simulations, stability and conformational behavior are typically assessed using RMSD, RMSF, radius of gyration, hydrogen-bond occupancy, interaction contact analysis, and secondary structure dynamics [132,133]. In addition, free-energy-based methods such as MM-PBSA and MM-GBSA are frequently used to estimate binding strength, whereas Ramachandran plots [134], free energy landscapes, and residue-level energy decomposition are applied to characterize structural quality and interaction mechanism [135].

Validation in structure-based studies is generally multi-stage. Computational screening is often supported by repeated simulations, consensus docking, and physicochemical filtering, including ADME assessment, Lipinski’s rule evaluation, and toxicity prediction [136]. Promising candidates are then tested experimentally using biochemical assays, cell-based assays, enzyme inhibition assays, IC_50_ measurements, or target-specific functional readouts. This workflow helps confirm that compounds selected in silico are not only structurally plausible binders but also biologically active under experimental conditions.

#### 3.1.2. Omics-Based

Omics-based methods rely on large-scale molecular and perturbation data to infer drug–disease relationships from transcriptional, genetic, or multi-omic signatures. Mendelian randomization (MR) studies commonly use human genetic resources such as UK Biobank and GWAS summary statistics [137,138], together with curated drug–target mappings from databases such as DrugBank and ChEMBL. Signature-based approaches often use Connectivity Map (CMap) and LINCS/L1000 to obtain drug-induced expression profiles [139,140], while GEO and TCGA are frequently used to derive disease-associated transcriptomic signatures [141,142]. Additional resources such as DrugBank, ChEMBL, GO, and ATC are often integrated to connect molecular signatures with mechanisms of action and clinical annotations.

The evaluation metrics used in omics-based methods depend on the specific analytical framework. For transcriptome reversal analysis, the key output is usually a reversal or connectivity score indicating whether a compound counteracts the disease signature. Enrichment-based measures, such as GSEA, are also used to assess the direction and strength of signature reversal [139]. For MR studies, effect estimates such as the Wald ratio and inverse-variance weighted (IVW) estimates are typically reported, together with heterogeneity tests and pleiotropy-sensitive models such as MR-Egger regression [143,144]. When omics signals are used to predict drug response, performance is often summarized using Spearman correlation, R^2^, or normalized AUC-based metrics.

Validation typically proceeds from retrospective confirmation to biological testing. Predicted candidates are first compared with known drug–disease associations or reported indications and then examined across independent datasets or external cohorts. MR-based studies often include sensitivity analyses, positive controls, and colocalization analyses to strengthen causal interpretation. For signature-based repurposing, validation may further involve in vitro experiments, the measurement of cell viability or cytopathic effects, and the verification of downstream molecular changes at the transcript or protein level [145]. In oncology and other disease settings, testing across multiple model systems is often required to distinguish genuine therapeutic activity from nonspecific effects.

#### 3.1.3. Fuzzy Logic-Based

Fuzzy logic-based methods integrate heterogeneous biomedical evidence by representing biological relationships in a graded rather than binary manner. These approaches commonly draw on databases such as DrugBank, KEGG, BRENDA, PDB, TCGA, DepMap, STRING, and GeneCards to obtain drug, protein, pathway, disease, and interaction information [125,126,131,142,146,147,148,149]. Compared with purely structure-based methods, fuzzy logic frameworks are particularly suited to combining partial evidence from genomic similarity, pathway association, phenotype similarity, and clinical knowledge within a unified inferential model.

Validation strategies in this class are typically layered. Most studies use cross-validation, often in the form of 10-fold cross-validation, to assess predictive robustness. Statistical tests such as the Wilcoxon signed-rank test, Friedman test, and Bonferroni correction are frequently applied to compare methods under different settings [150,151,152]. Biological validation is usually based on pathway consistency, protein–protein interaction support, cancer dependency evidence, mechanistic interpretation, or literature confirmation. In some cases, secondary computational checks, such as docking or network analysis, are also used to reinforce the plausibility of predicted candidates.

#### 3.1.4. Adverse Event-Based

Adverse event (AE)-based studies are built primarily on pharmacovigilance and biomedical reference resources. The most widely used source is the FDA Adverse Event Reporting System (FAERS), which provides spontaneous post-marketing reports for signal mining [153]. VigiBase is also frequently used to provide broader international reporting evidence, while MedDRA is commonly adopted for AE coding and phenotype standardization [154,155]. For drug annotation and mechanism interpretation, studies often integrate DrugBank, ATC, GEO, LINCS, ChEMBL, and ontology resources such as BioPortal [126,128,140,141,156,157].

Evaluation in AE-based studies focuses on signal strength and prioritization quality. The most common pharmacovigilance metrics are the reporting odds ratio (ROR), information component (IC), and empirical Bayes geometric mean (EBGM), which measure disproportionality between a drug and a specific adverse event [158,159,160]. When candidate ranking is involved, top-*k* measures and NDCG are often used to evaluate prioritization performance [161]. In integrative settings, cosine similarity, Jaccard similarity, reverse gene-expression scores, Spearman correlation, and AUROC are also used to compare AE-derived signals with disease-reversal or cluster-based patterns [162,163].

Validation usually extends beyond signal detection. A common strategy is retrospective recovery of known indication–drug pairs, or comparison with established therapeutic agents and curated database records [145]. Some studies also use leave-one-compound-out validation, external transcriptomic datasets, or independent pharmacological resources to test robustness [164]. Experimental confirmation may include cell viability assays, gene-expression readouts, animal studies, or retrospective clinical evidence. Additional practical filters, such as onset latency, package-insert information, and safety assessment, are often used to exclude implausible candidates and improve translational relevance.

### 3.2. Network-Based Methods

#### 3.2.1. Graph Mining

Graph mining-based methods, including graph clustering, random walk, graph diffusion, meta-path, and semantic-based approaches, typically rely on multi-relational biomedical networks assembled from curated databases. Common sources include DrugBank, OMIM, KEGG, CTD, CORUM, STRING, DisGeNET, GOA, ChEMBL, BindingDB, PharmGKB, and ClinicalTrials.gov, together with literature-curated associations [126,128,129,131,146,165,166,167,168,169,170,171]. These resources provide drug, disease, gene, pathway, phenotype, side-effect, and interaction information for constructing biologically meaningful graphs.

Evaluation metrics in graph mining-based studies depend on the task. For association prediction and ranking, AUROC and AUPR are the most frequently used metrics, with AUPR being particularly informative under class imbalance [163]. Many studies also report precision, recall, F1-score, MCC, specificity, sensitivity, and top-*k* hit rates. In graph clustering settings, modularity, cohesiveness, NMI, Jaccard similarity, and enrichment-based measures are often used to assess both topological coherence and biological relevance [161].

Validation is usually performed through internal resampling, external benchmarking, and retrospective evidence checking. Most studies adopt 5-fold or 10-fold cross-validation, whereas more stringent drug-wise or disease-wise splits are preferable when evaluating generalization to unseen cases. Predicted candidates are then compared against benchmark datasets, curated databases, or withheld known associations [172]. Additional support is often obtained from literature mining, disease-specific case studies, or independent experimental evidence when available [145].

#### 3.2.2. Matrix Factorization or Matrix Completion

Matrix factorization (MF) and matrix completion (MC) approaches are generally trained on curated drug–disease association matrices derived from resources such as DrugBank, OMIM, CTD, KEGG, repoDB [126,129,146,165,172], and benchmark datasets including PREDICT, LRSSL [55,173]. These datasets are often complemented by auxiliary similarity information, including chemical structure features, disease similarity derived from MeSH, UMLS, or Disease Ontology, side-effect profiles from SIDER, and target annotations from UniProt, SuperTarget, and related databases [174]. In this setting, the sparse interaction matrix serves as the core supervision signal, while side information helps regularize latent representations.

Performance is most commonly evaluated using AUROC and AUPR, reflecting the sparse and imbalanced nature of drug-disease association data. Additional metrics such as precision, recall, specificity, sensitivity, accuracy, and F1-score are also widely reported, and top-*k* precision or recall is frequently used to assess early enrichment of plausible candidates [163].

Validation is usually based on 5-fold or 10-fold cross-validation. To evaluate generalization to unseen drugs or diseases, some studies adopt de novo or cold-start settings in which one drug, one disease, or a subset of associations is withheld during training [164]. Candidate predictions are often further checked against updated external databases, clinical trial records, or literature evidence, and promising cases may be supported by case studies or domain-specific biological interpretation.

### 3.3. Data-Driven

#### 3.3.1. Text Mining-Based

Text mining-based studies primarily use biomedical literature and curated knowledge bases to extract relations between drugs, diseases, targets, and phenotypes. PubMed/MEDLINE and PMC are the most common text corpora [175,176], while DrugBank, CTD, TTD, UniProt, NCBI Gene, GO, KEGG, and PubChem provide structured annotations for relation normalization and downstream interpretation [126,127,130,146,165,177,178,179]. ClinicalTrials.gov and FDA-approved drug lists are also frequently used as external references for candidate prioritization and validation [171].

Evaluation typically relies on standard classification and ranking metrics, including precision, recall, F1-score, AUROC, AUPR, and MAP [163]. When models generate similarity scores or ranked candidate lists, Pearson and Spearman correlation coefficients are sometimes used to assess agreement with benchmark references or known rankings [161]. These metrics capture both relation extraction performance and the quality of candidate prioritization.

Validation generally combines internal and external evidence. Internal validation often includes k-fold cross-validation or chronological validation, whereas external validation is usually based on known drug indications, clinical trial records, FDA-approved drugs, or literature-supported evidence. In addition, manual case analysis and disease-specific expert review are frequently used to assess biological plausibility and practical relevance [145].

#### 3.3.2. Large Language Model-Based

Large language model (LLM)-based studies increasingly use biomedical literature, knowledge graphs, curated biomedical databases, and clinical records as input sources. Common resources include PubMed-derived corpora, GO, DrugBank, Hetionet, and task-specific benchmark datasets covering drug–target interactions, drug–disease associations, ADMET properties, and high-throughput screening data [17,130,175,180]. These resources provide complementary information on molecular mechanisms, drug properties, and clinical relevance, enabling LLMs to support hypothesis generation and candidate prioritization.

The choice of evaluation metric depends on the downstream task. For classification tasks such as drug–target interaction or drug–disease association prediction, AUROC/AUC, AUPR/AUPRC, precision, recall, F1-score, and specificity are most often reported [163]. For regression tasks, R^2^ is commonly used, whereas generative design tasks are typically assessed using validity, uniqueness, novelty, and diversity [181]. In clinically oriented studies, hazard ratio, confidence intervals, and *p*-values are used to quantify associations between predicted candidates and observed outcomes, and some agent-based frameworks additionally report valid rate.

Validation strategies range from retrospective benchmarking to real-world confirmation. Most studies rely on 5-fold cross-validation or repeated runs on benchmark datasets, while a smaller number use retrospective cohort analysis or external electronic health record data. Because LLMs may generate plausible but unverified outputs, manual expert review and hallucination checking are commonly used to filter candidates before downstream interpretation. Overall, the current LLM-based systems remain primarily hypothesis-generating tools, and their predictions still require orthogonal validation before translational use [145,182].

### 3.4. Summary

Across the three methodological paradigms, data sources, evaluation metrics, and validation strategies exhibit both convergence and divergence. All paradigms increasingly rely on common reference layers such as DrugBank, ChEMBL, and GO to anchor predictions, yet their primary evidence types differ fundamentally: biological mechanism-driven methods prioritize physicochemical and molecular data (structural coordinates, binding affinities, transcriptomic signatures); network-based methods center on relational and topological information (interaction graphs, association matrices); and data-driven methods, particularly LLM-based approaches, draw heavily on unstructured biomedical text and large-scale knowledge embeddings.

Consequently, evaluation metrics are largely incommensurable—physical plausibility is gauged by binding energy and conformational stability, whereas statistical and network methods predominantly report AUROC, AUPR, and ranking measures, and generative tasks adopt validity and novelty scores. These metrics assess distinct constructs and do not translate directly into clinical utility.

Validation strategies reveal a shared reliance on retrospective benchmarking against known drug–disease associations and k-fold cross-validation, but they diverge sharply in experimental depth. Structure-based and omics-based studies often advance to in vitro or in vivo confirmation, whereas network-based and purely data-driven studies remain largely confined to computational validation. Notably, LLM-based predictions currently depend almost entirely on manual expert review and hallucination checks, constituting the shortest validation chain among all paradigms. Despite occasional claims of clinical relevance, bona fide clinical validation, whether through prospective cohorts, randomized evidence, or real-world outcome tracking, remains exceedingly rare across all categories. This gap stems from structural barriers (high cost, lengthy timelines, regulatory complexity), methodological limitations (most predictions are correlative or hypothesis-generating rather than causally grounded), and incentive misalignments in academic evaluation that favor rapid computational publication over slow, resource-intensive translational follow-up. Until these barriers are addressed, the field will continue to produce a large volume of computationally promising candidates with limited clinically actionable evidence.

## 4. Discussions

### 4.1. Future Directions for LLM-Based Methods

Although LLM-based methods have demonstrated significant promise in semantic understanding, multi-source data integration, and complex task orchestration, their application in drug repurposing is still in its early stages. Challenges such as data bias, model hallucinations, and limited interpretability remain critical barriers. We propose the following three research directions as hypotheses to be tested and refined by future work.

First, a potential research direction is to explore whether LLMs can be enhanced to genuinely capture intrinsic biomedical knowledge. Current LLMs primarily rely on statistical co-occurrence patterns in text rather than a true understanding of biological mechanisms. One hypothesis is that integrating structured biomedical knowledge bases—such as Gene Ontology, signaling pathways, and protein–protein interaction networks—into the pre-training and inference processes of LLMs could enable models not only to “know what” but also to “understand why”, facilitating a transition from “language models” to more “knowledge-aware models.” However, the extent to which such integration improves factual reasoning and reduces domain-specific hallucinations remains an open question requiring systematic evaluation.

Second, another open question is how LLMs can be most effectively integrated with traditional methods. While LLMs excel at semantic representation, they often fall short in handling structured biomedical data such as molecular structures or protein interaction networks. We hypothesize that hybrid frameworks combining LLMs with structure-based methods, network analysis, matrix factorization, or graph neural networks could outperform either paradigm alone. For instance, LLMs might be used to extract implicit knowledge from literature, which is then validated through mechanistic models, forming a “semantics-guided, mechanism-driven” paradigm. Nevertheless, the optimal design of such hybrids—including where to place the integration point and how to balance computational costs—has yet to be determined, and future work should systematically benchmark them against traditional baselines.

Third, we hypothesize that human–AI collaborative validation frameworks, where experts interactively refine LLM-generated hypotheses, could enhance both reliability and practicality. Drug repurposing is a high-stakes endeavor that requires rigorous validation, and relying solely on model outputs poses substantial risks. Future systems could be designed to be interactive and interpretable, allowing researchers to intervene, refine, and validate hypotheses generated by LLMs. By integrating expert knowledge, experimental data, and clinical feedback, a closed-loop workflow of “model generation—expert review—experimental validation” may be established. The effectiveness of such frameworks relative to fully automated or fully manual approaches remains to be empirically tested, and factors such as workflow overhead, scalability, and generalizability across diseases require further investigation.

In summary, the future of drug repurposing research should not merely pursue larger models or higher performance metrics but, rather, emphasize the deep understanding of biomedical knowledge within models, their synergistic integration with traditional approaches, and effective collaboration with human experts, potentially achieving a paradigm shift from “data-driven” to “knowledge-driven” discovery. However, this vision requires sustained empirical validation and should be pursued as a set of testable hypotheses rather than as established conclusions.

### 4.2. A Proposed Framework of Drug Repurposing

A proposed framework of drug repurposing will be characterized by an intelligent, multi-stage integration of computational methodologies that leverage the strengths of each approach while mitigating their individual limitations. As outlined in this comprehensive review, we propose a three-phase paradigm that aligns with the evolving computational landscape and addresses the critical need for both efficiency and biological rigor.

Phase 1: High-throughput screening using network-based and data-driven methods.

The initial screening phase will increasingly rely on network-based and advanced data-driven methods, particularly LLM-based methods, for their unparalleled ability to integrate multi-source heterogeneous data and capture deep semantic relationships. These methods will serve as the primary engine for rapid hypothesis generation at scale, capable of processing millions of drug–disease interactions across diverse data modalities including genomic, transcriptomic, proteomic, and clinical data. Network-based methods will continue to play a foundational role in this phase by systematically uncovering latent associations through the global topology of biological networks. LLMs, particularly medical-specialized models, multi-source knowledge integration models and LLM agent collaborative models, excel at understanding contextual semantics and generating testable hypotheses from unstructured biomedical literature. These models can process complex relationships across multiple knowledge sources simultaneously, effectively transforming the initial screening phase from a labor-intensive process to a highly scalable, automated pipeline.

Phase 2: Mechanistic validation through biological mechanism-driven methods.

Following initial screening, the validation phase will focus on rigorous mechanistic elucidation using biological mechanism-driven methods. While network-based and data-driven methods excel at generating hypotheses, they cannot replace the need for understanding the underlying biological processes. This phase will require the application of structure-based, omics-based, fuzzy logic-based, and adverse event-based methods to validate the potential drug-disease relationships. Structure-based methods will provide atomic-level insights into drug–target interactions, molecular docking followed by MD simulations will confirm binding stability and specificity, and omics-based approaches will elucidate the molecular pathways affected by the drug. Fuzzy logic-based methods will offer a principled framework for handling the continuous nature of biological responses, while adverse event-based approaches will provide clinical context and safety signals. This mechanistic validation is critical for distinguishing true therapeutic effects from coincidental correlations.

Phase 3: Clinical translation and safety verification.

The final phase will involve clinical translation, where promising candidates undergo rigorous clinical trials to confirm efficacy and safety in the target population. This phase will increasingly incorporate real-world data and pharmacovigilance signals, particularly leveraging adverse event databases to identify potential safety concerns early. The integration of clinical trial data with computational predictions will create a continuous feedback loop, enabling iterative refinement of the computational methods. Promising candidates will proceed to clinical evaluation, yet this phase remains the most persistent bottleneck in the repurposing pipeline. While drug repurposing compresses the overall development timeline from the de novo average of 12–15 years to 3–12 years [4], the clinical segment alone, from first-in-human to successful Phase III publication, can still consume approximately 8 years, with oncology Phase III success rates hovering near 40% [183]. Regulatory agencies continue to demand robust monotherapy evidence and new dosing data, often requiring additional Phase I trials even for well-known drugs when used in new combinations or formulations. Concurrently, intellectual property barriers and weak market exclusivity for off-patent generics dismantle the commercial incentive to fund these trials, while academic sponsors struggle with protracted MTA negotiations and fragmented public funding. Pharmacologically, effective doses for the new indication frequently exceed original approvals, necessitating de novo PK/PD validation and extended safety surveillance in comorbid populations [184]. A representative end-to-end workflow [31] demonstrates what is possible when funding and regulatory coordination align, but it remains an exception rather than the norm. Ultimately, clinical outcomes must feed back into Phase I models to close the loop; however, the very delays that plague Phase III inevitably slow this iterative refinement, reminding us that computational acceleration at the front end is offset by procedural friction at the back end [185].

A plausible future direction is not the replacement of one paradigm by another but their staged integration into a closed-loop workflow. In such a framework, text mining-based and LLM-based methods can serve as semantic front ends for evidence extraction, literature synthesis, and hypothesis generation, while network-based methods can transform heterogeneous relations into graph- or matrix-level representations for large-scale prioritization. Promising candidates may then be subjected to biological mechanism-driven validation, including structure-based docking and molecular dynamics, omics-based signature reversal or causal inference, and adverse event-based or clinical corroboration. In parallel, LLMs may also facilitate cross-modal alignment by generating contextualized node features for graph learning, assisting rule construction in fuzzy logic-based modeling, and supporting human-in-the-loop validation. Such hybrid designs are likely to balance scalability, interpretability, and translational reliability more effectively than any single method family alone. Ultimately, the translational value of any computational repurposing framework is determined not by its in silico ranking performance alone but by whether its predictions can be confirmed in prospective clinical settings. Retrospective benchmarking, external database matching, and expert review are useful first steps, yet they cannot substitute for bona fide clinical validation through prospective cohorts, randomized evidence, or real-world outcome tracking. In this sense, network-based and data-driven methods should be regarded primarily as hypothesis-generating engines, whereas biological mechanism-driven methods provide an essential layer of mechanistic support; however, only clinical follow-up can establish whether a predicted association is truly actionable for patients. Despite encouraging recent examples, clinical validation remains exceedingly rare across current drug repurposing studies and continues to be the most persistent bottleneck in translational advancement.

## 5. Conclusions

This review has provided a comprehensive overview of the computational methodologies driving the field of drug repurposing. By systematically categorizing these methods, from the mechanistic insights offered by structure-based, omics-based, fuzzy logic-based, and adverse event-based methods, to the systems-level predictions enabled by network-based methods, and culminating in the advanced semantic understanding capabilities of text mining and large language model (LLM)-based methods, we have outlined a clear methodological evolution. This taxonomy, progressing from a focus on specific biological interactions to the integration of heterogeneous data and finally to the leveraging of vast textual knowledge, offers a logical framework for understanding the strengths and applicability of each paradigm. The primary significance of this methodological review lies in its systematic comparison. Each class of methods presents a unique set of advantages and inherent limitations. While structure-based methods provide deep mechanistic insights, they often depend on high-quality target protein structures. Omics-based methods can uncover novel disease–drug associations but may require robust validation to establish causality. Fuzzy logic-based methods transform qualitative intuition into computable operations through fuzzification, rule-based inference, and defuzzification, but they demand domain expertise for membership function design. Adverse event (AE)-based methods leverage real-world clinical observations as phenotypic signals, constrained by spontaneous reporting limitations. Network-based methods aim at integrating multi-source data for systematic prediction but can sometimes function as black boxes. Although traditional text mining unlocks valuable information from literature, its depth is limited. The emergence of LLMs represents a paradigm shift, offering an unprecedented ability to integrate multi-source data and capture deep semantic and contextual information. However, an overly enthusiastic attitude towards LLMs can lead to the risk of hallucinations. In conclusion, this review serves as a guide to the rapidly evolving computational landscape of drug repurposing. By clarifying the logical progression and interconnectedness of different methodologies, we hope to provide researchers with a valuable roadmap. As data resources expand and AI models become more powerful and interpretable, computationally driven drug repurposing is poised to play an increasingly central role in overcoming the traditional barriers of drug development, ultimately delivering safe and effective therapies to patients more rapidly and efficiently. Although each method family offers distinct advantages, all remain subject to limitations that must be carefully considered, including data incompleteness, limited interpretability, benchmark heterogeneity, and the need for rigorous experimental and clinical validation. By clarifying the strengths and limitations of current computational paradigms, we hope this review provides a useful reference for future methodological development and translational application in drug repurposing.

## Figures and Tables

**Table 1:** Comparative summary of computational methods for drug repurposing. Each method is characterized by techniques, key advantages, key limitations, and application scenarios.

Method	Techniques	Key Advantages	Key Limitations	Application Scenarios
Structure-based [8,10,15,18,19]	Molecular docking; molecular dynamics (MD) simulations	Atomic-level mechanistic insights; cost-effective candidate prioritization	Dependence on high-quality 3D target structures; high computational demands; scoring function approximations	Candidate target structures are available; complex stability validation; toxicity pathway identification
Omics-based [20,21,22,24,73]	Mendelian randomization (genomic data); signature-based approaches (transcriptomic data)	Mode of action (MoA); causal evidence	Dependence on strong cis-variants; cell death signals confound cancer screens; population bias	Causal inference for drug-disease relationships; mechanism of action elucidation
Fuzzy logic-based [25,26,27,28]	Fuzzy logic: fuzzification, rule-based inference and defuzzification	Transform qualitative concepts into computable operations; filter outliers without data resampling; interpretable and aligned with clinical reasoning	Domain expertise to define membership functions; manual definition of if-then rules	Side effect quantification via fuzzy equality operators; multi-objective optimization
Adverse event (AE)-based [29,30,31,32,33,74]	AE-primary; AE-auxiliary	Human-centered; scalable hypothesis generation	Spontaneous reporting biases; multi-metric assessments and orthogonal validation	Inverse phenotype identification; clinical feasibility; filter out the side-effect drugs
Network-based [35,39,42,45,50,54,57]	Graph clustering, random walk, graph diffusion, meta-path, semantic-based; matrix factorization or matrix completion	Integrate multi-source heterogeneous data; systems-level perspective on biological networks	Heavy dependence on data quality and completeness; cold-start problem; lack interpretability; difficulty distinguishing positive vs. negative drug-disease associations	Predict unknown drug-disease associations; integrate heterogeneous biomedical data; identify functional modules and therapeutic communities
Text mining-based [61,62,63,64,65,66,75]	Lexical co-occurrence; semantic parsing; logical reasoning; feature extraction; pre-trained language models	Detect direct and indirect relationships beyond manual curation; transform textual data into actionable knowledge; rapid retrieval and intuitive exploration	Potential source biases in literature; dependency on entity recognition quality; require careful validation of extracted relationships	Literature-based candidate identification; disease-specific knowledge graph construction; indirect relationship inference
LLM-based [67,68,69,70,71,72]	Medical specialized language models; multi-source knowledge integration models; LLM agent collaborative models	Superior contextual semantic understanding; zero-shot task adaptation and knowledge transfer; cross-modal knowledge integration	Model hallucinations generate biologically implausible predictions; heavy dependency on training data quality and completeness	Multi-source data integration; high-throughput screening; hypothesis generation and mechanistic inference

**Table 2:** Network-based methods using graph mining can be divided into graph clustering, random walks, graph diffusion, meta-path and semantic-based methods, according to different algorithms. All of them strive to predict the unknown drug-disease associations and have different limitations.

Methods	Key Idea	Limitations
Graph Clustering [35,36,37,87,88]	Direct Clustering:Use Louvain or ClusterONE to directly extract drug-disease associations Protein Complex-Mediated: Use protein complexes as functional bridges to infer drug-disease associations indirectly via a tripartite network where clustering validates predictions by grouping drugs with known therapeutics Network-derived Feature-Based: Combine network-derived features with classical clustering (e.g., K-means) to prioritize candidates grouping with approved drugs	Direct Clustering: Heavily rely on existing gene annotationsProtein Complex-Mediated: Difficulty distinguishing positive or negative associations Network-derived Feature-Based: Highly rely on feature quality
Random Walk [38,39,40,41]	Simulate a walker moving through a biological network, where the final probability of reaching a node reflects its therapeutic potential (e.g., bidirectional or dual-perspective walks, individualized walk lengths)	Heavily on data quality and parameter tuning; Lack interpretability
Graph Diffusion [42,43,44,89,90,91,92,93,94]	Non-parametric: Use graph diffusion directly on biological networks to predict drug-disease associations without learnable parameters Embedding learning integration: Employ graph diffusion as a feature-processing step within graph neural networks to capture long-range dependencies and learn powerful node embeddings for classification Hard negative sampling: Leverage a graph diffusion network to generate hard negative samples for contrastive learning, enhancing the discrimination of predictive models	Non-parametric: Highly dependent on prior knowledge and parameter tuning Embedding learning integration: Model complexity; Lack interpretability Hard negative sampling: Training and computational complexity
Meta-path [45,46,47,95,96]	Construct a biological network; define meaningful meta-paths (e.g., Drug → Protein → Disease) or employ graph learning techniques to automatically weight and aggregate information from multiple paths; use meta-paths as features for machine learning model	Initial reliance on domain expertise to predefine relevant meta-paths
Semantic-based [2,49,50,51,52,53,97]	Construct a semantic network that integrates diverse biological data (e.g., drugs, targets, diseases) using ontologies and formal logic, use semantically meaningful elements (e.g., specific path patterns or subgraphs that represent well-defined biological relationships) to mine this network	Dependence on ontology completeness and data quality; High computational complexity of formal reasoning; Limited adaptability due to predefined semantic rules

**Table 3:** Both Matrix factorization and matrix completion frame the drug repurposing as a recommendation system problem, but they have different ideas to predict missing/unknown items in the matrix.

Methods	Key Idea	Representative Methods	Prospects	Considerations
MF	Factorize drug-disease association matrix into a drug-feature matrix and a disease-feature matrix	DisDrugPred [56], MSBMF [59], SCMFDD [55], NTD-DR [60]	Interpretability	Cold-start problem, non-convex optimization
MC	Directly complete a low-rank matrix to approximate the known drug-disease association matrix	DRRS [54], OMC [57], DRIMC [58]	Alleviate cold-start problems	Computational complexity, dependence on similarity measures

## Data Availability

No new data were created or analyzed in this study. Data sharing is not applicable to this article.

## Abbreviations

The following abbreviations are used in this manuscript:

LLM(s)	Large language model(s)
MD	Molecular dynamics
MR	Mendelian randomization
AE	Adverse event
MF	Matrix factorization
MC	Matrix completion
